# Associations Between Both Smartphone Addiction and Objectively Measured Smartphone Use and Sleep Quality and Duration Among University Students: Cross-Sectional Study

**DOI:** 10.2196/77796

**Published:** 2025-11-25

**Authors:** Jian Yin, Xuanyi Tang, Zeshi Liu, Yangyang Gong, Hui Yang, Yanping Zhang

**Affiliations:** 1 Department of Laboratory, Second Affiliated Hospital of Xi'an Jiaotong University, No. 157 West Five Road, Xi'an, 710004, China, +86-15929443510; 2 School of Public Health, Shandong University, Jinan, China; 3 Department of External Cooperation and Exchange, Shaanxi Provincial Health Commission, Xi'an, China

**Keywords:** smartphone addiction, smartphone screen time, smartphone unlocks, sleep disorders, sleep duration

## Abstract

**Background:**

The impact of smartphone use on sleep remains intensely debated. Most existing studies have used self-reported smartphone use data. Moreover, few studies have simultaneously examined associations between both smartphone addiction and objectively measured smartphone use and sleep, and the dose-response relationship between smartphone use and risk of poor sleep has been consistently overlooked, requiring systematic and further research on this topic.

**Objective:**

This study aimed to examine the associations between smartphone addiction and objectively measured smartphone use and sleep quality and duration.

**Methods:**

This cross-sectional study enrolled 17,713 participants from a university in China. We assessed objective smartphone screen time and unlocks by collecting screenshots of use records and measured smartphone addiction using a validated questionnaire. Sleep quality and duration were estimated via the Pittsburgh Sleep Quality Index. Binary logistic regression, linear regression, and restricted cubic spline regression models were used for the analyses.

**Results:**

A total of 14.3% (2533/17,713) of the participants met the criterion for poor sleep, with a mean sleep duration of 507.1 (SD 103.2) minutes per night. Notably, university students with smartphone addiction exhibited 184% higher risk of poor sleep (odds ratio [OR] 2.84, 95% CI 2.59-3.11) and a 15.47-minute–shorter nighttime sleep duration (β*=*–15.47, 95% CI −18.53 to −12.42) compared to those without smartphone addiction. Regarding objectively measured smartphone use, participants with ≥63 hours per week of smartphone screen time had 22% higher odds of poor sleep (OR 1.22, 95% CI 1.08-1.37) and a 6.66-minute–shorter nighttime sleep duration (β*=*–6.66, 95% CI −10.19 to −3.13) compared to those with 0 to 21 hours of screen time per week, whereas those with approximately 21 to 42 hours per week of smartphone screen time had a 5.47-minute–longer nighttime sleep duration (β*=*5.47, 95% CI 1.28-9.65). Similarly, compared to those with 0 to 50 smartphone unlocks per week, participants with ≥400 smartphone unlocks per week showed 61% higher odds of poor sleep (OR 1.61, 95% CI 1.41-1.85) accompanied by a 4.09-minute–shorter nighttime sleep duration (β*=*–4.09, 95% CI −8.08 to −0.09), whereas those with approximately 50 to 150 smartphone unlocks per week had a 5.84-minute–longer sleep duration (β*=*5.84, 95% CI 2.32-9.36). An inverted U–shaped association between smartphone screen time and sleep duration was observed (*P*<.001 for nonlinearity).

**Conclusions:**

Smartphone addiction, excessive objectively measured smartphone screen time, and unlocks are positively associated with both sleep quality and duration. Restricted cubic spline analyses revealed different nuanced dose-response relationships, with an inverted U–shaped association observed between smartphone screen time and sleep duration.

## Introduction

In the digital age, youths spend more time on smartphones than ever before, and smartphones have become an inescapable part of everyday life [1]. Adequate and high-quality sleep plays a pivotal role in sustaining physiological and psychological well-being. Poor sleep quality alongside inadequate duration is both prevalent and strongly linked to elevated risks of adverse health outcomes [2-4] and, thus, has become a notable concern. Over recent decades, epidemiological evidence has indicated a progressive deterioration in both average sleep quality and duration [5], with roughly 33% of the global population falling short of internationally recommended nocturnal sleep thresholds (7‐9 hours per night). Given the public health impact of poor and insufficient sleep, there is a critical need to better understand the potential influence of ubiquitous smartphones in impairing sleep health.

Numerous studies have linked smartphone use with poor sleep and shorter sleep duration [6-12], whereas few studies have reported that smartphone screen time correlates with poorer sleep quality but has minimal effects on sleep duration [9,13]. Conversely, 2 studies found no association between the daily use of technology devices and sleep outcomes [14,15]. Notably, the current generation of youth, referred to as iGens by Twenge [16], has grown up in the smartphone era and is characterized by elevated rates of smartphone use and smartphone addiction [17]. University students today belong to the iGeneration, who are more prone to severe smartphone addiction and maladaptive use that may impair sleep quality and duration in the absence of familial oversight. This risk is particularly concerning because poor or insufficient sleep can exacerbate mental health issues, which have already garnered broad societal attention, especially against the backdrop of China’s rapidly accelerating pace of life. Previous research has posited that smartphones may affect sleep via multiple mechanisms. First, the time displacement hypothesis posits that excessive smartphone use potentially comes at the cost of sleep [18] as each additional hour of screen time theoretically reduces available sleep hours. A prospective cohort study showed that excessive smartphone use was related to shorter total sleep time in children [19]; the total sleep time of the smartphone overuse group (smartphone use of over 1 hour per day) was shorter than that of the control group. Second, prolonged smartphone screen time increases exposure to screen-emitted light [20]. More light exposure from smartphones could delay melatonin onset, disrupting the circadian rhythm and thereby reducing sleep quality and duration. Phillips et al [21] demonstrated that evening exposure to light at intensities below 30 lux, which is comparable to that emitted by smartphones, suppressed melatonin by 50%. Furthermore, the electromagnetic fields emitted by smartphones have also been identified as a significant factor influencing sleep. A study by Huber et al [22] revealed that exposure to pulse-modulated electromagnetic fields increased relative regional cerebral blood flow in the dorsolateral prefrontal cortex ipsilateral to the exposure and enhanced electroencephalographic power in the alpha frequency range before sleep onset and in the spindle frequency range during stage 2 sleep. Third, psychological and physical arousal induced by engaging, entertaining, or distressing content from various smartphone apps may also impair the ability to fall asleep and maintain sleep [23,24]. Meyerson et al [25] leveraged estimated bedtimes from 44,000 Reddit users and their 120 million posts, revealing that users who posted 4 or more times within 1 hour before bedtime had a 4-fold higher risk of delayed sleep onset compared to those who posted 2 to 5 hours before bedtime.

Despite a growing body of research on the association between smartphone use and sleep, methodological and dimensional limitations persist. First, the widespread use of smartphones in everyday life points to the need for rigorous and objective methods that can adequately capture the true links between smartphone use behavior and sleep. However, most existing studies rely on participants’ self-reported smartphone use data. This is an important limitation because previous research has shown that self-reported smartphone use is prone to recall bias and misclassification errors and does not correlate well with objectively measured smartphone use [26-29]. A neglected aspect of smartphone use is unlocks, defined as the number of times that users activate their smartphone screen within a specific period, a behavior reflecting compulsive urges for immediate interaction. As a dimension distinct from smartphone screen time, unlocks represent dynamic user-device interaction patterns [30,31] whose potential impact on sleep remains understudied. Second, research on the association between smartphone use and sleep has been siloed, focusing exclusively on either smartphone addiction or smartphone use (eg, duration). These 2 dimensions represent different metrics of smartphone use [32-34]. Smartphone addiction has been defined as an individual’s excessive and uncontrollable use of smartphones, resulting in impaired social functioning and psychological and behavioral problems [35,36]. Smartphone screen time is neither a necessary criterion nor a precise proxy for addiction as users with shorter smartphone screen time may still exhibit addictive symptoms, whereas those with longer screen time might engage in adaptive use [32]. Thus, analyzing smartphone addiction and smartphone use behaviors is essential to understanding the full impact of smartphones on sleep. To date, few studies have examined the associations between both smartphone addiction and smartphone use and sleep simultaneously. Moreover, previous research has often treated smartphone addiction and smartphone use behaviors as categorical variables in traditional regression models, thereby overlooking the potential for dose-response associations between continuous smartphone use and risk of poor sleep. Consequently, less is known about associations between different points along the continuum of both smartphone use and smartphone addiction and sleep quality or duration.

This large-scale study adopted a more holistic perspective with a representative sample, assessing objective smartphone use by collecting screenshots of participants’ smartphone screen time and unlock records and measuring smartphone addiction through a validated questionnaire, with the aim of examining whether smartphone addiction and objectively measured smartphone use were associated with sleep quality and duration among university students. We also conducted restricted cubic splines (RCS) regression to better understand the dose-response relationship between smartphone use and sleep.

## Methods

### Study Population

In this cross-sectional study, a university in Shaanxi province, China, was randomly selected from the provincial registry of higher-education institutions. All 24,156 students in this university were invited to participate in the survey. Before data collection, training sessions were conducted for all class counselors to explain the study objectives and procedures. They subsequently facilitated the anonymous completion of a structured electronic questionnaire by the students. From the 22,047 questionnaires received, submissions were excluded if they met any of the following criteria: (1) completion time of less than 500 seconds, (2) failure on one or more attention-check questions, (3) inability to provide valid smartphone use screenshots, or (4) implausible sleep metric data. Consequently, a total of 17,713 students were included in the final analyses. A flowchart of the screening of the study participants is shown in Multimedia Appendix 1.

### Ethical Considerations

The Second Affiliated Hospital of Xi’an Jiaotong University provided ethics approval for this study (approval ID: 2022-248). All participants gave their electronic informed consent before taking part in the study. All personally identifiable information was removed during data preparation and analysis and was not included in the manuscript or supplementary materials. No images or visual identifiers of participants are included in the study. Participants received no financial compensation for their involvement. This study followed the STROBE (Strengthening the Reporting of Observational Studies in Epidemiology) reporting guideline and CONSORT-EHEALTH (Consolidated Standards of Reporting Trials of Electronic and Mobile Health Applications and Online Telehealth) checklist [37]. The CONSORT-EHEALTH checklist is shown in Checklist 1.

### Measures

#### Smartphone Addiction

The Mobile Phone Addiction Tendency Scale (MPATS), originally developed by Xiong et al [38] for Chinese university populations, was used to evaluate smartphone addiction in this study. The scale comprises 16 items across 4 dimensions consisting of salience, withdrawal symptoms, social comfort, and mood changes. The items are scored on a scale from 1 (“very inconsistent”) to 5 (“very consistent”), yielding a total score between 16 and 80. Elevated total scores correlate with greater severity of smartphone addiction, with scores of ≥48 defining smartphone addiction. In this study, the Cronbach α coefficient was 0.94.

#### Objectively Measured Smartphone Use

The objectively measured smartphone use included smartphone screen time and unlocks. In this study, upon providing informed consent, participants were instructed to capture and submit screenshots of their smartphone use during the previous full week, which was automatically recorded by the built-in Screen Time tool of the iOS system and the Digital Wellbeing tool of the Android system. The operating system passively records these behavioral data through a triggering mechanism involving screen photoelectric sensors, representing an objective measurement approach independent of self-reporting. Previous studies have validated the use of such built-in tracking systems to obtain objective smartphone use behavior as accurate and reliable for capturing actual use patterns [29,39]. The 7-day assessment period aligns with those of most studies in this field [40-42]. There is evidence suggesting that less than 1 week of smartphone data collection is already sufficient to capture typical smartphone use behaviors [43]. Furthermore, to mitigate potential variation between weekdays and weekends and enhance the representativeness, participants were instructed to submit screenshots encompassing a full week (5 weekdays and 2 weekend days). Two weeks before the formal investigation of this study, we instructed the participants to activate the function for smartphone records to ensure the availability of objective data. We then provided step-by-step instructions for screenshots for different brands and types of smartphones and required them to submit the screenshots covering (1) smartphone screen time during the previous week and (2) number of smartphone unlocks during the previous week. According to the distribution of the screen time data, we classified participants into 4 groups (approximately 0-21, 21-42, 42-63, and ≥63 hours per week). In accordance with the distribution of our datasets, and to ensure better practical guidance, we partitioned smartphone unlocks into quartiles, selecting integer thresholds around quartile points. Consequently, we classified participants into 4 groups for smartphone unlocks (approximately 0-50, 50-150, 150-400, and ≥400 times per week).

#### Sleep Quality and Duration

The Pittsburgh Sleep Quality Index (PSQI) was administered to evaluate sleep quality over the previous month [44]. Comprising 19 items, the instrument is structured into 7 domains (subjective sleep quality, sleep latency, sleep duration, habitual sleep efficiency, sleep disturbances, use of sleeping medication, and daytime dysfunction). Each domain is scored on a scale between 0 and 3, with elevated scores representing poorer sleep. Aggregating scores across domains produces a composite PSQI score spanning 0 to 21, where higher scores signify poorer sleep quality. A score of ≥8 was used to define poor sleep in accordance with the validated cutoff for the Chinese population [45], which has demonstrated good reliability and validity in previous representative studies [46,47]. In this study, the Cronbach α coefficient of the PSQI was 0.85.

Sleep duration was evaluated via the following question: “Over the past month, how many hours did you typically spend asleep at night? This time should exclude time spent in bed without falling asleep.”

#### Covariates

We developed a structured questionnaire to gather several confounding factors. Sociodemographic information included gender, university year, race, registered permanent residence, whether the participants had siblings, and parental educational attainment. As described in a previous study [48], health-related lifestyles comprised current smoking, current drinking, physical activity, and rational diet. Participants who had smoked at least one cigarette in the previous 30 days were classified as current smokers. Similarly, those who had consumed at least one glass of wine during the previous 30 days were categorized as currently drinking. The International Physical Activity Questionnaire–Short Form was used to assess physical activity [49], categorizing activity levels into low, moderate, and high according to established calculated metabolic equivalents. An irrational diet was defined as participants consuming red meat every day or vegetables and fruits less than daily.

As previous studies have reported significant relationships between social support and sleep, the Adolescent Social Support Scale (ASSS) was administered to quantify social support [50]. The ASSS is a 16-item instrument, with each item scored on a scale from 1 to 5. A higher score signifies greater social support. The Cronbach α coefficient of the ASSS was 0.88, indicating strong internal consistency.

### Statistical Analyses

Participant characteristics were described using frequency distributions for categorical variables, as well as means and SDs for continuous variables. Three sets of binary logistic and linear regression models were constructed to estimate odds ratios and 95% CIs for poor sleep, as well as β coefficients and corresponding 95% CIs for sleep duration. Covariates included sex, university year, race, registered permanent residence, whether the participants had siblings, parental educational attainment, current smoking, current drinking, physical activity, rational diet, and social support. RCS regression was used to model dose-response associations between both smartphone addiction and objectively measured smartphone use and sleep quality and duration. In addition, we tested for interaction between smartphone use and sex to determine whether sex modified the relationships. To reduce the possible effects of the unbalanced sex ratio of the participants, we conducted a sex-weighted logistic regression. Moreover, a multilevel model with college under university as a random effect was conducted to mitigate the college under university clustering effects.

In all tests, a 2-sided *P* value of <.05 was used as a significance threshold. The R software (version 4.0.2; R Foundation for Statistical Computing) was used for all data analyses.

## Results

### Participant Characteristics

Table 1 presents the characteristics of the participants in this study. Of the 17,713 participants, 6087 (34.4%) were male, and 11,626 (65.6%) were female. A total of 95.9% (16,992/17,713) were of Han ethnicity, 43.1% (7633/17,713) lived in rural areas, and 34.4% (6098/17,713) were from single-child families. Notably, 14.3% (2533/17,713) of the participants met the criteria for poor sleep (PSQI≥8), with a mean sleep duration of 507.1 (SD 103.2) minutes per night. Overall, 24.9% (4418/17,713) of the participants met the criteria for smartphone addiction (MPATS score ≥48), with a mean MPATS score of 37.4 (SD 13.3). Mean smartphone screen time was 49.1 (SD 29.9) hours per week, and mean unlocks were 391.1 (SD 278.3) times per week.

**Table 1.** Characteristics of the participants (N=17,713).

Characteristic	Total participants (N=17,713)	Participants who did not meet the criteria for poor sleep (n=15,180)	Participants who met the criteria for poor sleep (n=2533)	*P* value
Sex, n (%)				<.001
Male	6087 (34.4)	5315 (87.3)	772 (12.7)	
Female	11,626 (65.6)	9865 (84.9)	1761 (15.1)	
University year, n (%)				<.001
First	6111 (34.5)	5256 (86.0)	855 (14.0)	
Second	4515 (25.5)	3803 (84.2)	712 (15.8)	
Third	4647 (26.2)	3959 (85.2)	688 (14.8)	
Fourth or above	2440 (13.8)	2162 (88.6)	278 (11.4)	
Ethnicity, n (%)				<.001
Han	16,992 (95.9)	14,599 (85.9)	2393 (14.1)	
Others	721 (4.1)	581 (80.6)	140 (19.4)	
Registered permanent residence, n (%)				.01
Rural	7633 (43.1)	6601 (86.5)	1032 (13.5)	
Urban	10,080 (56.9)	8579 (85.1)	1501 (14.9)	
Siblings, n (%)				.73
No	6098 (34.4)	5234 (85.8)	864 (14.2)	
Yes	11,615 (65.6)	9946 (85.6)	1669 (14.4)	
Maternal educational attainment, n (%)				.20
Middle school or lower	9915 (56.0)	8494 (85.7)	1421 (14.3)	
High school	4134 (23.3)	3572 (86.4)	562 (13.6)	
University or higher	3664 (20.7)	3114 (85.0)	550 (15.0)	
Paternal educational attainment, n (%)				.07
Middle school or lower	8600 (48.6)	7345 (85.4)	1255 (14.6)	
High school	4410 (24.9)	3826 (86.8)	584 (13.2)	
University or higher	4703 (26.6)	4009 (85.2)	694 (14.8)	
Current smoking, n (%)				<.001
No	14,599 (82.4)	12,747 (87.3)	1852 (12.7)	
Yes	3114 (17.6)	2433 (78.1)	681 (21.9)	
Current drinking, n (%)				<.001
No	13,369 (75.5)	11,826 (88.5)	1543 (11.5)	
Yes	4344 (24.5)	3354 (77.2)	990 (22.8)	
Rational diet, n (%)				<.001
Yes	2378 (13.4)	2162 (90.9)	216 (9.1)	
No	15,335 (86.6)	13,018 (84.9)	2317 (15.1)	
Physical exercise, n (%)				<.001
Moderate or high	3672 (20.7)	3247 (88.4)	425 (11.6)	
Low	14,041 (79.3)	11,933 (85.0)	2108 (15.0)	
Social support score, mean (SD, 16~80)	68.0 (15.2)	69.2 (14.9)	61.0 (14.7)	<.001
Smartphone addiction, n (%)				<.001
No	13,295 (75.1)	11,998 (90.2)	1297 (9.8)	
Yes	4418 (24.9)	3182 (72.0)	1236 (28.0)	
MPATS^a^ score, mean (SD, 16~80)	37.4 (13.3)	36.0 (12.7)	46.0 (13.3)	<.001
MPATS score—salience, mean (SD)	8.1 (3.3)	7.8 (3.2)	10.0 (3.6)	<.001
MPATS score—withdrawal symptoms, mean (SD)	15.3 (5.5)	15.4 (5.2)	18.7 (5.3)	<.001
MPATS score—social comfort, mean (SD)	7.2 (3.1)	6.9 (2.9)	8.8 (3.3)	<.001
MPATS score—mood changes, mean (SD)	6.8 (2.9)	6.5 (2.7)	8.5 (3.0)	<.001
Smartphone screen time (h per wk), n (%)				<.001
0-21	4117 (23.2)	3546 (86.1)	571 (13.9)	
21-42	2896 (16.3)	2570 (88.7)	326 (11.3)	
42-63	4588 (25.9)	3973 (86.6)	615 (13.4)	
≥63	6112 (34.5)	5091 (83.3)	1021 (16.7)	
Smartphone screen time (h per wk), mean (SD)	49.1 (29.9)	48.6 (29.6)	52.4 (31.0)	<.001
Smartphone unlocks (times per wk), n (%)				<.001
0-50	4062 (22.9)	3590 (88.4)	472 (11.6)	
50-150	5972 (33.7)	5195 (87.0)	777 (13.0)	
150-400	4249 (24.0)	3598 (84.7)	651 (15.3)	
≥400	3430 (19.4)	2797 (81.5)	633 (18.5)	
Smartphone unlocks (times per wk), mean (SD)	391.1 (278.3)	384.4 (274.2)	425.5 (296.4)	<.001

a MPATS: Mobile Phone Addiction Tendency Scale.

### Association Between Smartphone Use and Sleep Quality

**Figure 1.**
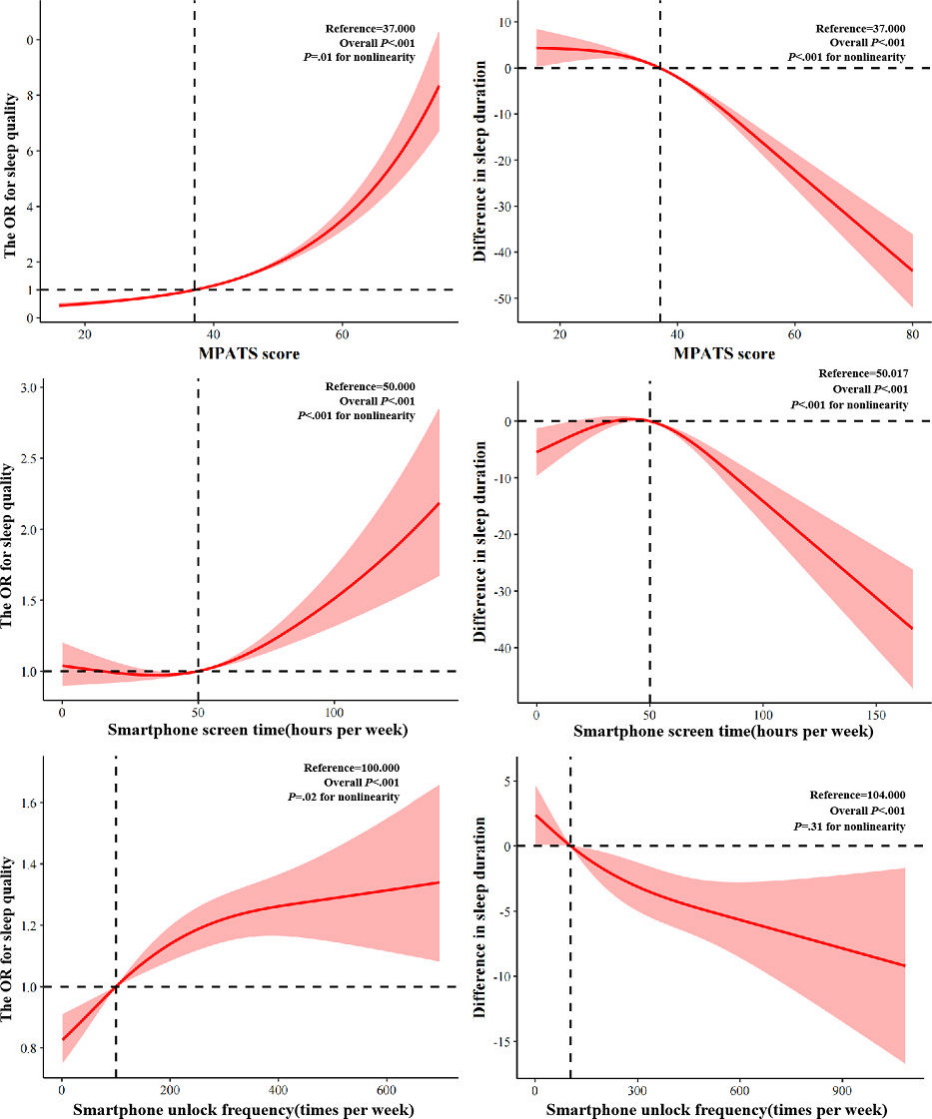
Restricted cubic spline regression analyses of the association between smartphone addiction, smartphone screen time, and smartphone unlocks and risk of poor sleep quality and reduction in sleep duration. These analyses were adjusted for sex, university year, race, registered permanent residence, whether the participants had siblings, parental educational attainment, current smoking, current drinking, physical activity, rational diet, and social support. MPATS: Mobile Phone Addiction Tendency Scale; OR: odds ratio.

The associations of smartphone addiction, screen time, and unlocks with sleep quality were presented in (Table 2). After adjusting for sex, university year, race, registered permanent residence, whether the participants had siblings, parental educational attainment, current smoking, current drinking, physical activity, rational diet, and social support, smartphone addiction was associated with poor sleep (OR 2.84, 95% CI 2.59-3.11). A 5-point increase in the MPATS score was substantially associated with poor sleep (OR 1.27, 95% CI 1.25-1.30). Objectively measured smartphone use was also linked to sleep quality. Compared with participants reporting 0–21 hours/week of smartphone screen time, those with ≥63 hours/week had higher odds of poor sleep (OR 1.22, 95% CI 1.08-1.37). While those with 21–42 hour/week (OR 0.86, 95% CI 0.74-1.00) and 42-63 hour/week (OR 0.98, 95% CI 0.86-1.12) exhibited no significant increase in sleep quality. Compared with participants reporting 0-50 times/week of unlocks, those with 150~400 times/week (OR 1.34, 95% CI 1.18-1.53) and ≥400 times/week had higher poor sleep odds (OR 1.61, 95% CI 1.41-1.85), while those with 50-150 times/week (OR 1.11, 95% CI 0.97-1.26) exhibited no change in sleep duration. A 21-hour/week increase in smartphone screen time (OR 1.08, 95% CI 1.04-1.11) and a 50 times/week increase in unlocks (OR 1.03, 95% CI 1.02-1.04*)* were associated with poor sleep. In models 1 and 2, the ORs increased slightly. Additionally, RCS analyses suggested nonlinear associations of MPATS score (*P=*.01 for nonlinearity), smartphone screen time (*P*<.001 for nonlinearity), and unlocks (*P*=.02 for nonlinearity) with the increasing risk of poor sleep (Figure 1).

**Table 2.** Associations between smartphone addiction, smartphone screen time, and smartphone unlocks and sleep quality and duration.

Smartphone use variable	Poor sleep*,* OR^a^ (95% CI)			Sleep duration, β (95% CI)		
	Model 1^b^	Model 2^c^	Model 3^d^	Model 1	Model 2	Model 3
Smartphone addiction						
No	Reference	Reference	Reference	Reference	Reference	Reference
Yes	3.54 (3.25 to 3.87)	3.20 (2.93 to 3.50)	2.84 (2.59 to 3.11)	−19.19 (−22.19 to –16.20)	−17.99 (−21.01 to –14.96)	−15.47 (−18.53 to –12.42)
MPATS^e^ score—sleep per-5 score	1.33 (1.31 to 1.36)	1.31 (1.28 to 1.33)	1.27 (1.25 to 1.30)	−3.93 (−4.42 to –3.44)	−3.72 (−4.22 to –3.22)	−3.20 (−3.71 to –2.69)
MPATS score—salience	1.21 (1.19 to 1.22)	1.19 (1.17 to 1.21)	1.17 (1.15 to 1.18)	−2.58 (−2.98 to –2.19)	−2.41 (−2.80 to –2.01)	−1.98 (−2.39 to –1.57)
MPATS score—withdrawal symptoms	1.14 (1.13 to 1.15)	1.13 (1.12 to 1.14)	1.12 (1.11 to 1.13)	−1.73 (−1.97 to –1.49)	−1.62 (−1.87 to –1.38)	−1.39 (−1.64 to –1.15)
MPATS score—social comfort	1.21 (1.19 to 1.23)	1.20 (1.18 to 1.22)	1.17 (1.16 to 1.19)	−3.25 (−3.68 to –2.83)	−3.09 (−3.53 to –2.67)	−2.65 (−3.09 to –2.20)
MPATS score—mood changes	1.27 (1.26 to 1.29)	1.25 (1.23 to 1.27)	1.23 (1.21 to 1.24)	−3.37 (−3.82 to –2.91)	−3.17 (−3.63 to –2.71)	−2.74 (−3.21 to –2.27)
Smartphone screen time (h per wk)						
0-21	Reference	Reference	Reference	Reference	Reference	Reference
21-42	0.79 (0.69 to 0.92)	0.84 (0.72 to 0.96)	0.86 (0.74 to 1.00)	6.19 (1.99 to 10.39)	5.71 (1.52 to 9.91)	5.47 (1.28 to 9.65)
42-63	0.97 (0.85 to 1.09)	0.97 (0.85 to 1.09)	0.98 (0.86 to 1.12)	3.48 (−0.25 to 7.21)	3.37 (−0.36 to 7.11)	3.42 (−0.30 to 7.14)
≥63	1.22 (1.09 to 1.37)	1.19 (1.06 to 1.34)	1.22 (1.08 to 1.37)	−7.07 (−10.60 to –3.53)	−6.86 (−10.40 to –3.32)	−6.66 (−10.19 to –3.13)
*P* value for the trend	<.001	<.001	<.001	<.001	<.001	<.001
Smartphone screen time increase of 21 h per week	1.09 (1.05 to 1.12)	1.08 (1.04 to 1.11)	1.08 (1.04 to 1.11)	−2.21 (−3.14 to –1.28)	−2.12 (−3.05 to –1.19)	−1.99 (−2.93 to –1.07)
Smartphone unlocks (times per wk)						
0-50	Reference	Reference	Reference	Reference	Reference	Reference
50-150	1.14 (1.01 to 1.29)	1.08 (0.95 to 1.22)	1.11 (0.97 to 1.26)	5.69 (2.16 to 9.22)	5.86 (2.33 to 9.39)	5.84 (2.32 to 9.36)
150-400	1.38 (1.22 to 1.57)	1.31 (1.15 to 1.50)	1.34 (1.18 to 1.53)	−3.75 (−7.56 to 0.06)	−3.44 (−7.25 to 0.37)	−3.28 (−7.08 to 0.52)
≥400	1.74 (1.53 to 1.98)	1.54 (1.35 to 1.76)	1.61 (1.41 to 1.85)	−4.79 (−8.77 to –0.79)	−4.05 (−8.06 to –0.04)	−4.09 (−8.08 to –0.09)
*P* value for the trend	<.001	<.001	<.001	<.001	<.001	<.001
Smartphone unlocks per 50 times per wk	1.04 (1.03 to 1.04)	1.03 (1.02 to 1.04)	1.03 (1.02 to 1.04)	−0.53 (−0.78 to –0.28)	−0.49 (−0.74 to –0.23)	−0.49 (−0.74 to –0.23)

a OR: odds ratio.

b Adjusted for sex, university year, race, registered permanent residence, whether the participants had siblings, and parental educational attainment.

c Adjusted for sex, university year, race, registered permanent residence, whether the participants had siblings, parental educational attainment, current smoking, current drinking, physical activity, and rational diet.

d Adjusted for sex, university year, race, registered permanent residence, whether the participants had siblings, parental educational attainment, current smoking, current drinking, physical activity, rational diet, and social support.

e MPATS: Mobile Phone Addiction Tendency Scale.

#### Association Between Smartphone Use and Sleep Duration

In model 3 in Table 2, smartphone addiction remained substantially associated with shorter sleep duration (β*=*–15.47, 95% CI −18.53 to −12.42). For objectively measured smartphone use, compared with participants reporting 0 to 21 hours per week of smartphone screen time, those with ≥63 hours per week had a shorter sleep duration (β*=*–6.66, 95% CI −10.19 to −3.13), whereas 21 to 42 hours of screen time per week (β=5.47, 95% CI 1.28-9.65) were linked to longer sleep duration, and those with 42 to 63 hours of screen time per week (β=3.42, 95% CI −0.30 to 7.14) showed no significant association with sleep duration. Similarly, compared to those with approximately 0 to 50 smartphone unlocks per week, those with ≥400 unlocks per week had a significantly shorter sleep duration (β*=*–4.09, 95% CI −8.08 to −0.0), and participants with 50 to 150 smartphone unlocks per week had a longer sleep duration (β*=*5.84, 95% CI 2.32-9.36). No substantial increase in sleep duration (β*=*–3.28, 95% CI −7.07 to 0.52) was observed for those with approximately 150 to 400 unlocks per week. Notably, each 5-point increase in the MPATS score (β*=*–3.20, 95% CI −3.71 to −2.69), 21–hour per week increase in smartphone screen time (β*=*–1.99, 95% CI −2.93 to −1.07), and 50–time per week increase in unlocks (β*=*–0.49, 95% CI −0.74 to −0.23) was substantially negatively associated with sleep duration. In models 1 and 2, the β coefficients were not changed materially. The results from the sex-weighted logistic regression model (Multimedia Appendix 2) and multilevel model with college as a random effect (Multimedia Appendix 3) were consistent with the primary findings, showing no significant changes. The RCS analyses suggested positive nonlinear associations between increasing MPATS score and shortened sleep duration (*P*<.001 for nonlinearity). What is more, an inverted U–shape nonlinear relationship was observed between smartphone screen time and reduction in sleep duration (*P*<.001 for nonlinearity), whereas increasing smartphone unlocks were found to be associated with monotonically decreasing sleep duration (*P*=.31 for nonlinearity; Figure 1).

### Sex-Stratified Analyses

The results of the sex-stratified analyses are shown in Table 3. This study found significant sex-specific differences in the relationships between smartphone screen time (*P=*.01 for the interaction) and unlocks (*P=*.002 for the interaction) and sleep duration. Notably, a 21–hour per week increase in smartphone screen time (β*=*–0.13, 95% CI 0.19 to −0.08) and a 50–time per week increase in unlocks (β*=*–0.77, 95% CI −1.08 to −0.47) were significantly associated with sleep duration in female individuals but not significant in male individuals. No significant sex-specific differences were observed in the relationships between smartphone addiction and sleep quality and duration (*P*>.05 for the interaction in all cases), nor were there significant associations between smartphone screen time and unlocks and sleep quality (*P*>.05 for the interaction in all cases; Table 3).

**Table 3.** Sex-specific associations between smartphone addiction, smartphone screen time, and smartphone unlocks and sleep quality and duration

Smartphone use variable^a^	Poor sleep*,* OR^b^ (95% CI)		*P* value for the interaction of smartphone use and sex on sleep	Sleep duration, β (95% CI)		*P* value for the interaction
	Male individuals	Female individuals		Male individuals	Female individuals	
Smartphone addiction			.70			.60
No	Reference	Reference		Reference	Reference	
Yes	2.74 (2.32 to 3.22)	2.89 (2.59 to 3.23)		−16.18 (−21.80 to −10.55)	−14.74 (−18.36 to −11.13)	
MPATS^c^ score per-5	1.27 (1.23 to 1.31)	1.28 (1.25 to 1.31)	.70	−3.07 (−3.96 to −2.17)	−3.19 (−3.82 to −2.57)	.74
MPATS score—salience	1.12 (1.10 to 1.14)	1.12 (1.11 to 1.13)	.96	−1.38 (−1.82 to −0.95)	−1.36 (−1.66 to −1.07)	.86
MPATS score—withdrawal symptoms	1.18 (1.15 to 1.20)	1.16 (1.14 to 1.18)	.46	−2.13 (−2.83 to −1.43)	−1.81 (−2.31 to −1.31)	.51
MPATS score—social comfort	1.19 (1.16 to 1.22)	1.17 (1.15 to 1.19)	.33	−2.58 (−3.39 to −1.77)	−2.67 (−3.18 to −2.15)	.94
MPATS score—mood changes	1.22 (1.19 to 1.26)	1.23 (1.20 to 1.25)	.87	−2.62 (−3.47 to −1.76)	−2.70 (−3.27 to −2.14)	.81
Smartphone screen time (h per wk)			.57			.22
0-21	Reference	Reference		Reference	Reference	
21-42	0.87 (0.68 to 1.19)	0.87 (0.72 to 1.05)		7.00 (−0.13 to 14.13)	4.06 (−1.11 to 9.23)	
42-63	0.88 (0.69 to 1.10)	1.02 (0.87 to 1.19)		7.33 (0.78 to 13.89)	0.91 (−3.61 to 5.42)	
≥63	1.10 (0.97 to 1.46)	1.24 (1.07 to 1.43)		−2.97 (−9.27 to 3.34)	−8.88 (−13.13 to −4.62)	
*P* value for the trend	.09	<.001		.39	<.001	
Smartphone screen time per 21 h per wk	1.00 (1.00 to 1.01)	1.00 (1.00 to 1.01)	.31	−0.03 (−0.10 to 0.05)	−0.13 (−0.19 to −0.08)	.01
Smartphone unlocks (times per wk)			.56			.001
0-50	Reference	Reference		Reference	Reference	
50-150	1.07 (0.85 to 1.35)	1.12 (0.97 to 1.31)		13.66 (7.26 to 20.06)	1.89 (–2.26 to 6.04)	
150-400	1.40 (1.10 to 1.79)	1.32 (1.13 to 1.55)		3.54 (−3.45 to 10.54)	−6.73 (−11.18 to −2.27)	
≥400	1.50 (1.17 to 1.93)	1.69 (1.43 to 1.98)		5.68 (−1.78 to 13.15)	−8.99 (−13.72 to −4.27)	
*P* value for the trend	<.001	<.001	—^d^	.59	<.001	—
Smartphone unlocks per 50 times per wk	1.03 (1.01 to 1.04)	1.03 (1.02 to 1.04)	.30	−0.03 (–0.48 to 0.42)	−0.77 (−1.08 to −0.46)	.002

a Adjusted for university year, race, registered permanent residence, whether the participants had siblings, parental educational attainment, current smoking, current drinking, physical activity, rational diet, and social support).

b OR: odds ratio.

c MPATS: Mobile Phone Addiction Tendency Scale.

d Not applicable.

## Discussion

### Principal Findings

This study systematically revealed the diverse associations between both smartphone addiction and objectively measured smartphone use and sleep among university students. Specifically, we found significant negative associations between smartphone addiction and both sleep quality and duration. Excessive objectively measured screen time and unlocks were significantly and positively associated with poor sleep and shorter sleep duration.

### Comparison With Other Studies

Despite the growing number of studies on the association between smartphone use and sleep, previous research has been mainly siloed, focusing exclusively on the effect of either smartphone addiction or objectively measured smartphone use on sleep quality. For instance, a systematic review revealed a significantly increased risk of poor sleep in individuals with smartphone addiction [51]. In a complementary nationally representative twin study, self-reported problematic digital technology use remained significantly correlated with poor sleep after accounting for confounders [52]. More recently, a study among university students uncovered positive associations between smartphone addiction and both bedtime procrastination and poor sleep [53]. Kaya et al [7] reported a significant relationship between self-reported smartphone use and poor sleep quality among university students using the Smartphone Addiction Scale–Short Version to assess smartphone addiction. Another study showed that smartphone addiction was linked to sleep quality and sleep duration [54]. The results of this study on the association between smartphone addiction and sleep are consistent with the findings of these studies.

In recent years, studies have increasingly focused on associations between smartphone use behaviors and sleep outcomes, yet reported findings remain inconsistent. For instance, a small-sample study using daily self-report smartphone use data found no association between smartphone talk time or screen time and sleep quality and duration [15]. In contrast, a larger study among adults revealed that self-reported daily smartphone use time was linked to poorer sleep quality, whereas unlocks showed no association with sleep parameters [55]. However, most existing research relies on self-reported smartphone use data, which are susceptible to recall bias and misclassification. The inconsistencies across findings highlight the critical need for more objective measures of smartphone screen time and unlocks to capture the true associations between smartphone use and sleep. To date, few studies have leveraged objectively measured smartphone use data to explore associations with sleep [13,56,57], and no consensus has been reached regarding these relationships. A study directly measured smartphone screen time in 136 participants via a tracking app and found that longer smartphone screen time was associated with poorer sleep efficiency and shorter sleep duration [56]. A Chinese study tracked participants’ smartphone use using the same method and indicated that each minute of daytime smartphone use was related to a 0.07-minute reduction in same-night total sleep time but unrelated to sleep efficiency and postsleep awakenings [57]. Only 1 recent study identified smartphone screen time and unlocks as significant predictors of total sleep time, albeit concluding that this effect was minimal [13]. All 3 of these studies concur that excessive objectively measured smartphone use reduces sleep duration while showing less consistency in their findings regarding the impact on sleep quality, particularly sleep efficiency. Notably, our study confirmed that excessive smartphone screen time and unlocks were associated with reductions in sleep duration by 6.66 minutes and 9.90 minutes per night, respectively, as well as an increased risk of poor sleep.

Novelly, an inverted U–shaped nonlinear association was observed between smartphone screen time and reduction in sleep duration. The inverted U–shaped relationship was matched by a corresponding delay in sleep offset. No previous studies have focused on the dose-response relationship between smartphone use and sleep, although some support can be drawn from several studies on presleep smartphone use. For example, a study that objectively assessed smartphone use before sleep using a tracking app found that using smartphones before sleep for longer than usual was associated with sleeping earlier and sleeping longer among adolescents [58], suggesting a potentially positive effect on sleep duration. A recent study showed that total screen use in the 2 hours before bedtime was associated with delayed sleep onset but not with total sleep time [9]. Similarly, Exelmans and Van den Bulck [59] suggested that bedtime smartphone use predicted later rise times, which may indicate a compensation behavior for missed sleep. Collectively, these 3 studies suggest that the impact of smartphone use on sleep duration may be less pronounced than that shown in some studies based on self-reported smartphone use data. Our study further identified an inverted U–shaped relationship between average smartphone use time and sleep duration via RCS regression, providing an important contribution to the existing literature. However, as this was a cross-sectional study that assessed associations at a single time point, longitudinal cohort research is critical to validate causality and assess long-term trajectories.

In this study, the β coefficient for the association between both smartphone screen time and unlocks and sleep duration was more pronounced in female than in male individuals. Sex differences in the association may stem from sex-specific preferences for smartphone apps. Compared with male individuals, female individuals exhibit stronger preferences for social media platforms [60], which are characterized by high unlock frequencies. Social media platforms are highly interactive, marked by frequent user engagement. The captivating content on these platforms is more likely to induce emotional arousal and subsequent sleep onset delay. This study did not evaluate sex-specific app preferences among students. Hence, future research should gather detailed data on the use of apps to further explore sex-specific differences and underlying mechanisms.

### Possible Explanations of the Associations

Several reasons may underlie the association between smartphone use and sleep. The reduction in sleep duration among university students with high smartphone use levels can be mechanistically explained through several pathways. The negative impact of smartphone use on sleep duration is consistent with the time displacement hypothesis [18], whereas the impairments in sleep quality can be explained by the combined effects of screen-emitted light on melatonin suppression [20] and psychological arousal from engaging content [23,24]. Furthermore, the association between smartphone addiction and sleep may additionally involve individuals’ negative cognitive appraisals of their use, such as perceived loss of control over smartphone use and guilt regarding excessive use, which may exacerbate sleep impairment. However, the reduction in sleep duration observed among students with low smartphone use levels represents a key novelty of this study, and it may be attributed to the following mechanisms. First, from a psychosocial perspective, smartphones have become highly integrated into the social interactions and academic activities of university students. Excessively low smartphone use may indicate potential difficulties in social connection or emotion regulation for individuals [61], and these factors are established risk factors for sleep, thus accounting for the observed reduction in sleep duration in the context of low smartphone use. Second, from a theoretical standpoint, the conservation of resources theory posits that technologies such as smartphones play a crucial role in the acquisition and maintenance of psychosocial resources [62]. Two studies even showed that smartphone nonusers are generally more prone to experiencing depression and anxiety [63,64]. Another study measured objective smartphone use in the context of the relationship between smartphone use and well-being, showing that people with intermediate smartphone use had better mood and lower loneliness than those with low smartphone use [65]. Third, in terms of sleep behavior patterns, individuals with low smartphone use may exhibit an earlier sleep phase, especially for those who rise early, which can result in objectively shorter total sleep duration. An objective measurement study using a wearable sleep and activity tracker, smartphone-delivered ecological momentary assessments, and passive smartphone use tracking also reported a similar pattern [65], finding that individuals with low bedtime smartphone use had earlier wake times (9:25 AM; –1 hour, 30 minutes to +1 hour, 30 minutes) than high bedtime smartphone users (10:08 AM; –1 hour, 49 minutes to +1 hour, 49 minutes).

### Limitations

First, although the models included a wide range of demographic and lifestyle factors, some confounding variables may have remained unaccounted for, potentially influencing the study findings. Second, sleep quality and duration were assessed using a retrospective self-report questionnaire, which may result in recall bias. Future studies would benefit from incorporating objective sleep measurements to triangulate the findings. Third, due to the cross-sectional study design, causal inferences regarding the relationship between smartphone use and sleep cannot be made. Future longitudinal studies are warranted to address it. Fourth, the generalizability of our findings may be limited by the exclusive focus on university students in China and the uneven sex distribution in the study sample. Fifth, although this study objectively measured smartphone use time and frequency via smartphone screenshot, data in this study still lack granularity regarding temporal dynamics (eg, distribution over the day or week) and functional variation (eg, social media, entertainment, education, or work). These details may be important for understanding the nuanced associations between smartphone use and sleep. Sixth, although the multilevel model treating class membership as a random effect yielded substantially unchanged results, this study did not account for clustering effects stemming from shared living or social environments (eg, classes, dormitories, and clubs). Future research could address these clustering effects by implementing simple random sampling methods, which would help minimize potential biases arising from nonindependent observations.

### Conclusions

This study found significant associations between smartphone addiction, excessive objectively measured screen time, and unlocks and both poor sleep and shorter sleep duration. RCS analyses revealed different nuanced dose-response relationships, with an inverted U–shaped association observed between smartphone screen time and sleep duration.

## Supplementary material

10.2196/77796 Multimedia Appendix 1 Flowchart of screening study participants.

10.2196/77796 Multimedia Appendix 2 Associations between smartphone addiction, smartphone screen time, and smartphone unlocks and sleep quality and duration in the sex-weighted model.

10.2196/77796 Multimedia Appendix 3 Associations between smartphone addiction, smartphone screen time, and smartphone unlocks and sleep quality and duration in the multilevel model with college under university as a random effect.

10.2196/77796 Checklist 1 CONSORT-EHEALTH checklist.

## Abbreviations

ASSS: Adolescent Social Support Scale
CONSORT-EHEALTH: Consolidated Standards of Reporting Trials of Electronic and Mobile Health Applications and Online Telehealth
MPATS: Mobile Phone Addiction Tendency Scale
OR: odds ratio
PSQI: Pittsburgh Sleep Quality Index
RCS: restricted cubic splines
STROBE: Strengthening the Reporting of Observational Studies in Epidemiology

## References

[1] Hartstein LE, Mathew GM, Reichenberger DA (2024). The impact of screen use on sleep health across the lifespan: a National Sleep Foundation consensus statement. Sleep Health.

[2] Sun L, Li K, Zhang Y, Zhang L (2022). Differentiating the associations between sleep quality and suicide behaviors: a population-based study in China. J Affect Disord.

[3] Gueye-Ndiaye S, Redline S (2025). Sleep health disparities. Annu Rev Med.

[4] Morales-Ghinaglia N, Fernandez-Mendoza J (2023). Sleep variability and regularity as contributors to obesity and cardiometabolic health in adolescence. Obesity (Silver Spring).

[5] Ford ES, Cunningham TJ, Croft JB (2015). Trends in self-reported sleep duration among US adults from 1985 to 2012. Sleep.

[6] Zhang J, Zhang X, Zhang K (2022). An updated of meta-analysis on the relationship between mobile phone addiction and sleep disorder. J Affect Disord.

[7] Kaya F, Bostanci Daştan N, Durar E (2021). Smart phone usage, sleep quality and depression in university students. Int J Soc Psychiatry.

[8] Xie YJ, Cheung DS, Loke AY (2020). Relationships between the usage of televisions, computers, and mobile phones and the quality of sleep in a Chinese population: community-based cross-sectional study. J Med Internet Res.

[9] Brosnan B, Haszard JJ, Meredith-Jones KA, Wickham SR, Galland BC, Taylor RW (2024). Screen use at bedtime and sleep duration and quality among youths. JAMA Pediatr.

[10] Li Y, Chen Q, He M (2024). Investigation of bi-directional relations between pre-sleep electronic media use and sleep: a seven-day dairy study. Comput Human Behav.

[11] Drews HJ, Sejling C, Andersen TO, Varga TV, Jensen AK, Rod NH (2024). Tracked and self-reported nighttime smartphone use, general health, and healthcare utilization: results from the SmartSleep Study. Sleep.

[12] Han X, Zhou E, Liu D (2024). Electronic media use and sleep quality: updated systematic review and meta-analysis. J Med Internet Res.

[13] Heneghan C, McDuff D, Winbush A (2024). 0313 Analysis of objective and subjective sleep metrics and smartphone usage patterns. Sleep.

[14] Cabré-Riera A, van Wel L, Liorni I (2022). Estimated all-day and evening whole-brain radiofrequency electromagnetic fields doses, and sleep in preadolescents. Environ Res.

[15] Eeftens M, Pujol S, Klaiber A (2023). The association between real-life markers of phone use and cognitive performance, health-related quality of life and sleep. Environ Res.

[16] Twenge JM (2017). Igen: Why Today’s Super-Connected Kids Are Growing Up Less Rebellious, More Tolerant, Less Happy--and Completely Unprepared for Adulthood--and What That Means for the Rest of Us.

[17] Thapa K, Lama S, Pokharel R, Sigdel R, Rimal SP (2020). Mobile phone dependence among undergraduate students of a medical college of eastern Nepal: a descriptive cross-sectional study. J Nepal Med Assoc.

[18] Kraut R, Patterson M, Lundmark V, Kiesler S, Mukopadhyay T, Scherlis W (1998). Internet paradox. A social technology that reduces social involvement and psychological well-being?. Am Psychol.

[19] Kim SY, Han S, Park EJ (2020). The relationship between smartphone overuse and sleep in younger children: a prospective cohort study. J Clin Sleep Med.

[20] Xu YX, Zhang JH, Tao FB, Sun Y (2023). Association between exposure to light at night (LAN) and sleep problems: a systematic review and meta-analysis of observational studies. Sci Total Environ.

[21] Phillips AJ, Vidafar P, Burns AC (2019). High sensitivity and interindividual variability in the response of the human circadian system to evening light. Proc Natl Acad Sci U S A.

[22] Huber R, Treyer V, Borbély AA (2002). Electromagnetic fields, such as those from mobile phones, alter regional cerebral blood flow and sleep and waking EEG. J Sleep Res.

[23] Carter B, Rees P, Hale L, Bhattacharjee D, Paradkar MS (2016). Association between portable screen-based media device access or use and sleep outcomes: a systematic review and meta-analysis. JAMA Pediatr.

[24] Hale L, Dzierzewski JM (2024). Screens and sleep health-it’s almost bedtime, time to put your phone away. JAMA Pediatr.

[25] Meyerson WU, Fineberg SK, Andrade FC, Corlett P, Gerstein MB, Hoyle RH (2023). The association between evening social media use and delayed sleep may be causal: suggestive evidence from 120 million Reddit timestamps. Sleep Med.

[26] Molaib KM, Sun X, Ram N, Reeves B, Robinson TN (2025). Agreement between self-reported and objectively measured smartphone use among adolescents and adults. Comput Hum Behav Reports.

[27] Mahalingham T, McEvoy PM, Clarke PJ (2023). Assessing the validity of self-report social media use: evidence of no relationship with objective smartphone use. Comput Human Behav.

[28] Mireku MO, Mueller W, Fleming C (2018). Total recall in the SCAMP cohort: validation of self-reported mobile phone use in the smartphone era. Environ Res.

[29] Ryding FC, Kuss DJ (2020). Passive objective measures in the assessment of problematic smartphone use: a systematic review. Addict Behav Rep.

[30] Qirtas MM, Zafeiridi E, White EB, Pesch D (2023). The relationship between loneliness and depression among college students: mining data derived from passive sensing. Digit Health.

[31] Wang W, Wu M, Yuan X (2025). Objectively measured smartphone use and nonsuicidal self-injury among college students: cross-sectional study. JMIR Ment Health.

[32] Sohn SY, Krasnoff L, Rees P, Kalk NJ, Carter B (2021). The association between smartphone addiction and sleep: a UK cross-sectional study of young adults. Front Psychiatry.

[33] Kalk N, Carter B, Sohn SY (2021). Are smartphones addictive? An urgent question in the pandemic age. Health Policy Technol.

[34] Panova T, Carbonell X (2018). Is smartphone addiction really an addiction?. J Behav Addict.

[35] Griffiths M (1996). Gambling on the internet: a brief note. J Gambling Stud.

[36] Lin YH, Chiang CL, Lin PH (2016). Proposed diagnostic criteria for smartphone addiction. PLoS ONE.

[37] Eysenbach G, CONSORT-EHEALTH Group (2011). CONSORT-EHEALTH: improving and standardizing evaluation reports of web-based and mobile health interventions. J Med Internet Res.

[38] Xiong J, Zhou ZK, Chen W, You ZQ, Zhai ZY (2012). Development of mobile phone addiction tendency scale for college students. China J Ment Health.

[39] Cao J, Truong AL, Banu S, Shah AA, Sabharwal A, Moukaddam N (2020). Tracking and predicting depressive symptoms of adolescents using smartphone-based self-reports, parental evaluations, and passive phone sensor data: development and usability study. JMIR Ment Health.

[40] Elhai JD, Tiamiyu MF, Weeks JW, Levine JC, Picard KJ, Hall BJ (2018). Depression and emotion regulation predict objective smartphone use measured over one week. Pers Individ Dif.

[41] Prasad S, Harshe D, Kaur N (2018). A study of magnitude and psychological correlates of smartphone use in medical students: a pilot study with a novel telemetric approach. Indian J Psychol Med.

[42] Rozgonjuk D, Levine JC, Hall BJ, Elhai JD (2018). The association between problematic smartphone use, depression and anxiety symptom severity, and objectively measured smartphone use over one week. Comput Human Behav.

[43] Wilcockson TD, Ellis DA, Shaw H (2018). Determining typical smartphone usage: what data do we need?. Cyberpsychol Behav Soc Netw.

[44] Buysse DJ, Reynolds CF 3rd, Monk TH, Berman SR, Kupfer DJ (1989). The Pittsburgh Sleep Quality Index: a new instrument for psychiatric practice and research. Psychiatry Res.

[45] Liu xianchen TM, Lei H, Aizhen W, Hongxin W (1996). Reliability and validity of the Pittsburgh Sleep Quality Index [Article in Chinese]. Chin J Psychiatry.

[46] Zhang S, Zhao Y, Qin Z (2024). Transcutaneous auricular vagus nerve stimulation for chronic insomnia disorder: a randomized clinical trial. JAMA Netw Open.

[47] Huang Q, Li Y, Huang S (2020). Smartphone use and sleep quality in Chinese college students: a preliminary study. Front Psychiatry.

[48] Yang G, Cao X, Li X (2022). Association of unhealthy lifestyle and childhood adversity with acceleration of aging among UK Biobank participants. JAMA Netw Open.

[49] Lee PH, Macfarlane DJ, Lam TH, Stewart SM (2011). Validity of the International Physical Activity Questionnaire Short Form (IPAQ-SF): a systematic review. Int J Behav Nutr Phys Act.

[50] Dai XY (2008). Development of social support scale for university students. Chin J Clin Psychol.

[51] Yang J, Fu X, Liao X, Li Y (2020). Association of problematic smartphone use with poor sleep quality, depression, and anxiety: a systematic review and meta-analysis. Psychiatry Res.

[52] Madrid-Valero JJ, Matthews T, Barclay NL (2023). Problematic technology use and sleep quality in young adulthood: novel insights from a nationally representative twin study. Sleep.

[53] Huang T, Liu Y, Tan TC, Wang D, Zheng K, Liu W (2023). Mobile phone dependency and sleep quality in college students during COVID-19 outbreak: the mediating role of bedtime procrastination and fear of missing out. BMC Public Health.

[54] Uzunçakmak T, Ayaz-Alkaya S, Akca A (2022). Prevalence and predisposing factors of smartphone addiction, sleep quality and daytime sleepiness of nursing students: a cross-sectional design. Nurse Educ Pract.

[55] Dissing AS, Andersen TO, Nørup LN, Clark A, Nejsum M, Rod NH (2021). Daytime and nighttime smartphone use: a study of associations between multidimensional smartphone behaviours and sleep among 24,856 Danish adults. J Sleep Res.

[56] Christensen MA, Bettencourt L, Kaye L (2016). Direct measurements of smartphone screen-time: relationships with demographics and sleep. PLoS ONE.

[57] Lee PH, Tse AC, Wu CS, Mak YW, Lee U (2021). Temporal association between objectively measured smartphone usage, sleep quality and physical activity among Chinese adolescents and young adults. J Sleep Res.

[58] Tkaczyk M, Lacko D, Elavsky S, Tancoš M, Smahel D (2023). Are smartphones detrimental to adolescent sleep? An electronic diary study of evening smartphone use and sleep. Comput Human Behav.

[59] Exelmans L, Van den Bulck J (2016). Bedtime mobile phone use and sleep in adults. Soc Sci Med.

[60] Gaya AR, Brum R, Brites K (2023). Electronic device and social network use and sleep outcomes among adolescents: the EHDLA study. BMC Public Health.

[61] Buda TS, Khwaja M, Garriga R, Matic A (2023). Two edges of the screen: unpacking positive and negative associations between phone use in everyday contexts and subjective well-being. PLoS ONE.

[62] Hobfoll SE (1989). Conservation of resources: a new attempt at conceptualizing stress. Am Psychol.

[63] Studer J, Marmet S, Wicki M, Khazaal Y, Gmel G (2022). Associations between smartphone use and mental health and well-being among young Swiss men. J Psychiatr Res.

[64] Xin M, Mo PK, Li J (2022). Smartphone non-users experience disproportionately higher psychological distress than their counterparts: mediations via psychosocial resources in a large sample of college students in China. J Affect Disord.

[65] Massar SA, Ng AS, Soon CS (2022). Reopening after lockdown: the influence of working-from-home and digital device use on sleep, physical activity, and wellbeing following COVID-19 lockdown and reopening. Sleep.

