# Supplementary figures and images for: Associations Between Both Smartphone Addiction and Objectively Measured Smartphone Use and Sleep Quality and Duration Among University Students: Cross-Sectional Study
Source: JMIR Ment Health. 2025 Nov 25;12:e77796. doi: 10.2196/77796 (PMC12646561; doi:10.2196/77796)

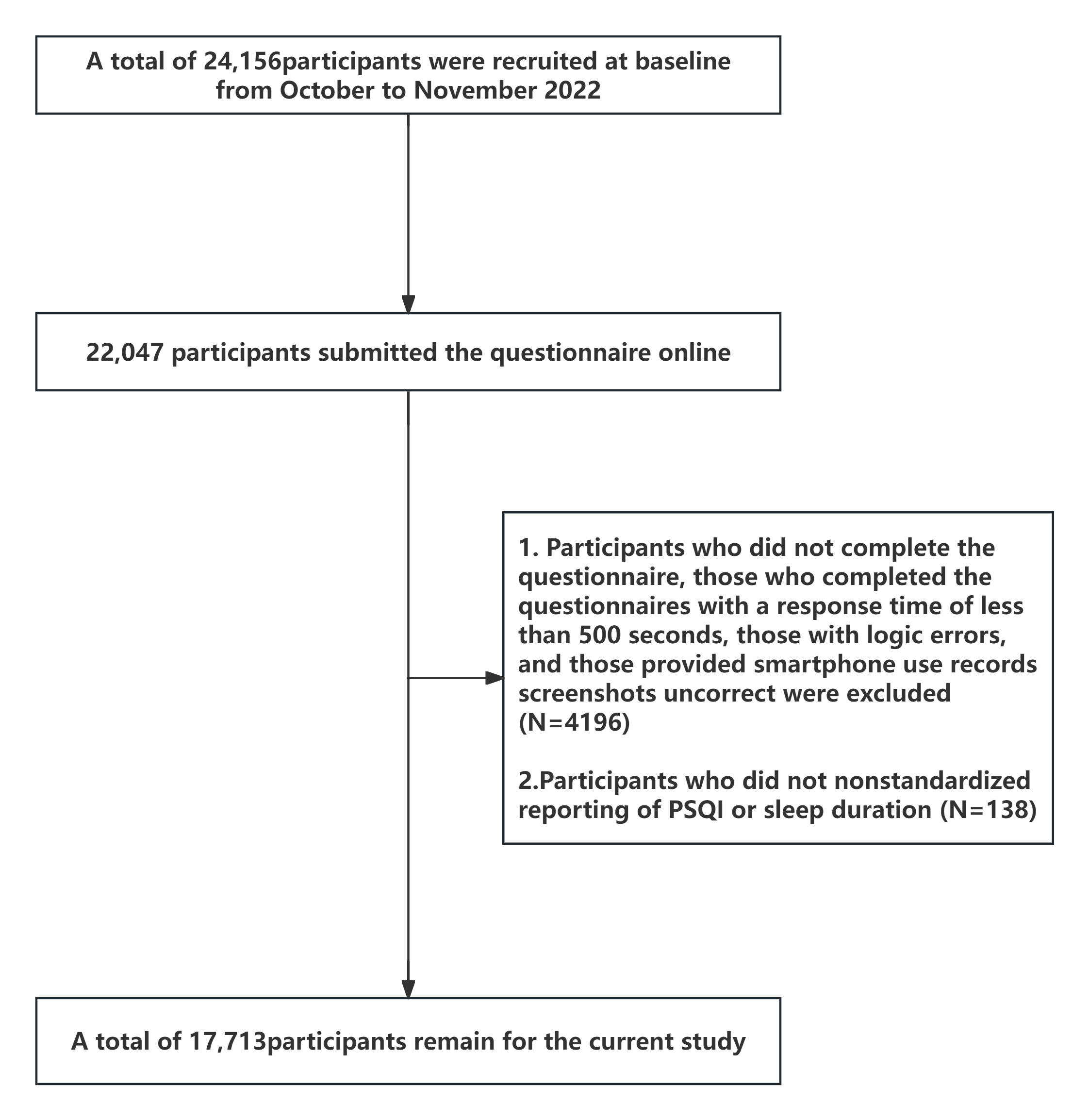

Supplement: Multimedia Appendix 1 [file mental-v12-e77796-s001.png]
