# Supplementary material for: Associations Between Both Smartphone Addiction and Objectively Measured Smartphone Use and Sleep Quality and Duration Among University Students: Cross-Sectional Study
Source: JMIR Ment Health. 2025 Nov 25;12:e77796. doi: 10.2196/77796 (PMC12646561; doi:10.2196/77796)
Supplement: Multimedia Appendix 3 [file mental-v12-e77796-s003.docx]

**Table S2. Associations of smartphone addiction, smartphone screen time, and smartphone unlocks with sleep quality and sleep duration by multilevel model with college as a random effect.**

| Smartphone use | Poor sleep*, OR (95%CI)* | | | Sleep duration, *β (95%CI)* | | |
| --- | --- | --- | --- | --- | --- | --- |
|  | Model 1 | Model 2 | Model 3 | Model 1 | Model 2 | Model 3 |
| Smartphone addiction |  |  |  |  |  |  |
| No | Reference | Reference | Reference | Reference | Reference | Reference |
| Yes | 3.60 (3.20, 4.05) | 3.24 (2.94, 3.58) | 2.89 (2.55, 3.28) | -18.82 (-24.15, -13.48) | -17.69 (-23.05, -12.33) | -15.12 (-20.51, -9.74) |
| MPATS score, mean (SD) per-5 | 1.33 (1.31, 1.36) | 1.31 (1.28, 1.33) | 1.28 (1.25, 1.30) | -3.79 (-4.67, -2.91) | -3.59 (-4.47, -2.71) | -3.06 (-3.95, -2.17) |
| MPATS- salience, mean (SD) | 1.14 (1.13, 1.15) | 1.13 (1.12, 1.14) | 1.12 (1.11, 1.13) | -1.08 (-2.54, 0.38) | -1.55 (-2.07, -1.03) | -0.75 (-2.23, 0.73) |
| MPATS-withdrawal symptoms, mean (SD) | 1.21 (1.19, 1.23) | 1.19 (1.18, 1.21) | 1.17 (1.15, 1.19) | -2.54 (-3.01, -2.07) | -2.36 (-2.91, -1.82) | -1.94 (-2.40, -1.48) |
| MPATS-social comfort, mean (SD) | 1.21 (1.19, 1.23) | 1.21 (1.19, 1.23) | 1.18 (1.16, 1.20) | -3.21 (-3.80, -2.61) | -3.06 (-3.67, -2.46) | -2.61 (-3.17, -2.04) |
| MPATS-mood changes, mean (SD) | 1.27 (1.25, 1.29) | 1.25 (1.23, 1.27) | 1.23 (1.21, 1.25) | -3.25 (-3.86, -2.65) | -3.01 (-3.87, -2.15) | -2.56 (-3.43, -1.69) |
| Smartphone screen time (hour/week) |  |  |  |  |  |  |
| 0~21 | Reference | Reference | Reference | Reference | Reference | Reference |
| 21~42 | 0.81 (0.69, 0.95) | 0.84 (0.72, 0.99) | 0.89 (0.74, 1.06) | 6.42 (-0.12, 12.95) | 5.94 (-0.52, 12.39) | 5.65 (-0.74, 12.04) |
| 42~63 | 0.96 (0.84, 1.09) | 0.95 (0.84, 1.08) | 0.98 (0.85, 1.13) | 3.52 (-1.63, 8.67) | 3.41 (-1.72, 8.54) | 3.41 (-1.59, 8.42) |
| ≥63 | 1.23 (1.09, 1.39) | 1.20 (1.06, 1.36) | 1.24 (1.07, 1.44) | -6.92 (-11.74, -2.10) | -6.70 (-11.61, -1.79) | -6.53 (-11.39, -1.68) |
| *P_trend_* | <0.001 | <0.001 | <0.001 | <0.001 | <0.001 | <0.001 |
| Smartphone screen time, per 21 hour/week | 1.09 (1.05, 1.14) | 1.08 (1.04, 1.11) | 1.08 (1.04, 1.11) | -2.11 (-3.75, -0.47) | -2.02 (-3.66, -0.37) | -1.90 (-3.55, -0.26) |
| Smartphone unlocks (times/week) |  |  |  |  |  |  |
| 0~50 | Reference | Reference | Reference | Reference | Reference | Reference |
| 50~150 | 1.14 (1.00, 1.29) | 1.11 (0.95, 1.29) | 1.14 (0.98, 1.32) | 5.57 (2.11, 9.03) | 5.49 (1.82, 9.16) | 5.35 (1.47, 9.23) |
| 150~400 | 1.42 (1.22, 1.64) | 1.38 (1.15, 1.66) | 1.41 (1.17, 1.70) | -3.37 (-6.87, 0.13) | -3.28 (-6.97, 0.41) | -3.23(-6.92, 0.46) |
| ≥400 | 1.72 (1.49, 2.00) | 1.57 (1.34, 1.84) | 1.65 (1.41, 1.92) | -4.37 (-8.15, -0.59) | -4.25 (-8.31, -0.19) | -4.20 (-8.20,-0.10) |
| *P_trend_* | <0.001 | <0.001 | <0.001 | <0.001 | <0.001 | <0.001 |
| Smartphone unlocks, per 50 times/week | 1.04 (1.03, 1.04) | 1.03 (1.02, 1.04) | 1.03 (1.02, 1.04) | -0.54 (-0.81, -0.27) | -0.50 (-0.76, -0.23) | -0.50 (-0.77, -0.23) |

Note: Model 1, adjusted for gender, grade, race, registered permanent residence, siblings, and parental educational attainment. Model 2, adjusted for gender, grade, race, registered permanent residence, siblings, parental educational attainment, current smoking, current drinking, physical activity, and rational diet. Model 3 adjusted for gender, grade, race, registered permanent residence, siblings, parental educational attainment, current smoking, current drinking, physical activity, rational diet, and social support.
